# Health communication implications of the perceived meanings of terms used to denote unhealthy foods

**DOI:** 10.1186/s40608-016-0142-0

**Published:** 2017-01-10

**Authors:** Simone Pettigrew, Zenobia Talati, Iain S. Pratt

**Affiliations:** 1 School of Psychology and Speech Pathology, Curtin University Kent St, Bentley, 6102 WA Australia; 2 Cancer Council Western Australia, 420 Bagot Rd, Subiaco, 6008 WA Australia

**Keywords:** Junk food, EDNP foods, Nutrition guidelines, Health promotion

## Abstract

**Background:**

Using appropriate terminology in nutrition education programs and behaviour change campaigns is important to optimise the effectiveness of these efforts. To inform future communications on the topic of healthy eating, this study explored adults’ perceptions of the meaning of four terms used to describe unhealthy foods: junk food, snack food, party food, and discretionary food.

**Methods:**

Australian adults were recruited to participate in an online survey that included demographic items and open-ended questions relating to perceptions of the four terms. In total, 409 respondents aged 25–64 years completed the survey.

**Results:**

‘Junk food’ was the term most clearly aligned with unhealthiness, and is therefore likely to represent wording that will have salience and relevance to many target audience members. Snack foods were considered to include both healthy and unhealthy food products, and both snack foods and party foods were often described as being consumed in small portions. Despite being used in dietary guidelines, the term ‘discretionary food’ was unfamiliar to many respondents.

**Conclusions:**

These results demonstrate that different terms for unhealthy foods can have substantially different meanings for audience members. A detailed understanding of these meanings is needed to ensure that nutrition guidance and health promotion campaigns use appropriate terminology.

## Background

Food marketers invest substantially in creative and pervasive advertisements to encourage wide-spread consumption of unhealthy foods [1–3]. These foods are defined in the literature as being energy dense and nutrient poor (EDNP) [4]. Given highly constrained budgets, the public health sector has to compete with this well-resourced marketing activity in the most effective and efficient manner possible [5]. This entails carefully crafting nutrition-related messages to ensure they convey clear, accurate, and persuasive information to target audiences [6, 7]. In particular, it is important that the terms used to describe EDNP foods are aligned with common language usage to ensure target segments understand the types of foods that are being referred to in the messages [8].

Numerous terms exist for EDNP foods, including unhealthy food, fast food, junk food, discretionary food, convenience food, party food, extra foods, treats, and snacks [9, 10]. Although many of these terms are used interchangeably, it is likely that they have somewhat different meanings for different people, and there may also be variations by population sub-segments (e.g., age, gender, and weight status). Understanding these subtle differences in meaning can potentially assist social marketers develop nutrition advertising campaigns that ‘ring true’, and are therefore more effective in terms of awareness, comprehension, and behavioural change [8, 11].

The objective of the present study was to identify key differences in Australian adults’ understanding of four nutrition-related terms to inform future campaigns designed to encourage reduced intake of EDNP foods. The four terms were ‘junk food’, ‘party food’, ‘snack food’, and ‘discretionary food’. The first three terms are commonly colloquially used in Australia, while ‘discretionary food’ reflects the terminology used in the Australian Healthy Eating Guidelines [12]. A multi-method dimensional analysis approach was adopted to provide insight into the complex meanings assigned to these common terms.

## Methods

Respondents were recruited to participate in an online survey via a large web panel provider. The survey instrument included demographic items and four open-ended questions that asked respondents to explain what the four terms meant to them (e.g., “What does the term “junk food” mean to you?”). The eligibility criteria were being a resident of Western Australia and aged between 25 and 64 years. The resulting sample comprised 409 adults, two-thirds (63%) of whom were female and one-third (34%) male (2% did not report their gender). Approximately one-quarter of respondents fell into each of the following age ranges: 25–34 (24%), 35–44 (25%), 45–54 (26%), and 55–64 (23%). Two percent did not report their age. In terms of body mass index (BMI: calculated by self-reported height and weight data), 37% of the sample was under/normal weight, 30% overweight, and 30% obese (3% did not provide height and/or weight data). The Australian adult prevalence rates are 37, 35, and 28% respectively [13].

Reflecting the nature of the data, both quantitative and qualitative analyses were conducted. This involved initially importing the responses to the short-answer questions into NVivo11, where they were coded according to the themes that emerged and frequency counts were conducted using NVivo’s matrix search function. Where feasible, chi-square tests were then conducted to test for any differences by gender, age, and BMI. Analyses were feasible where the total number of respondents in a cell was greater than 20 and the expected frequency was greater than two [14, 15].

## Results

According to the responses provided, the four terms included in this study were perceived to vary on several key attributes. These attributes and their frequency of mention across the four terms are shown in Table 1, with significant differences noted (at *p* < .05). Overall, relatively few significant demographic differences were found, indicating similar interpretations of the terms across population segments.

**Table 1:** Number of respondents nominating characteristics of each food term (*n* = 408)

	Health concerns			Consumption patterns				Preparation factors		
	General unhealthiness	Specific negative nutrients	Need for moderation	Portion size	Between meals	Fingers	Special events	Convenient	Processed/pre-packaged	Take away/QSR^a^
Junk food	207^cd^	108	9	2	0	0	0	28	37	54
Snack food	27	9	1	99^c^	163^d^	1	6	63	11	1
Party food	30	20	10	33	0	69^d^	91^c^	25^c^	5	0
Discretionary food^b^	18	7	60^e^	1	1	0	1	0	0	3

^a^ *QSR* quick service restaurants

^b^273 (67%) respondents indicated they were unaware of the meaning of this term or provided an incorrect description

^c^Significantly higher (*p* < .05) among younger (under 35) than older (over 55) participants

^d^Significantly higher (*p* < .05) among females than males

^e^Significantly higher (*p* < .05) among normal weight than overweight and obese participants

As shown in Table 1, the various attributes mentioned by respondents were collapsed into three primary dimensions that appeared to be most indicative of perceptions of the four terms. These dimensions were ‘health concerns’, ‘consumption patterns’, and ‘preparation factors’. Responses relating to health concerns included comments about the foods being generally ‘bad for you’, containing high levels of negative nutrients, and requiring moderation. Responses relating to consumption patterns were those that referred to the manner in which the food was eaten (in small portion sizes, between meals, with fingers, or at special events), and responses allocated to the preparation factors dimension were those relating to the convenience with which the foods could be purchased and served.

**Fig. 1:**
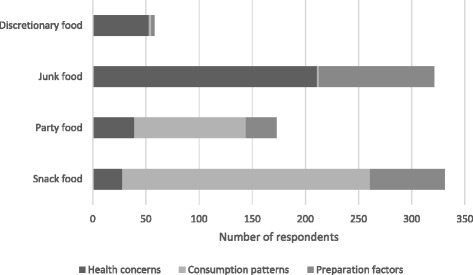
Dimensional analysis of respondents’ perceptions of food terms

Figure 1 illustrates the proportional influence of each dimension on respondents’ overall reactions to each food term. This analysis is useful in demonstrating both the extent and nature of responses elicited by each term. In the findings outlined below, indicative quotes demonstrate the respondents’ perceptions of each term, with demographic descriptors provided.

One of the four terms stood out as being markedly different from the others due to high levels of respondent confusion. Two-thirds (67%) of the sample indicated they did not understand the meaning of the term ‘discretionary foods’. This confusion was apparent across all demographic categories:

*Have not heard this term before (F, 25–34, normal-weight).*

*I’ve never heard the term and I have no idea (F, 35–44, obese).*

*No idea, never heard of it (M, 25–34, obese).*

*I have never heard this term (M, 50–64, obese).*

Among those who did exhibit some appreciation of the meaning of this term, there was a tendency to refer to concepts relating to choice and decision-making. As shown in Fig. 1, the decision-making process typically involved active consideration of the unhealthiness of these kinds of foods. Among normal weight respondents in particular, there was acknowledgement of the need to consume discretionary foods in moderation. This was the only attribute on which there was a difference in responses according to weight status.

*You can eat but only now and again, at your discretion (F, 45–54, normal-weight)*

*Food that you know is a bit naughty but you eat in moderation (F, 25–34, normal-weight)*

*Food you have to think about that are not healthy food choices but may be eaten from time to time (F, 55–64, obese)*

*Something you have the power to decide whether you eat or not (M, 25–34, normal-weight)*

Other respondents appeared to attempt to guess the meaning of the term:

*Food people deny publicly eating (M, 25–34, obese)*

*Not sure. A particular type of food based on religion –* e.g.*, Halal or if you are a Vegan, food containing no meat products (F, 55–64, normal-weight)*

The primary focus of respondents’ reactions to the term ‘snack food’ was on factors associated with how the foods are consumed rather than their nutritional content (Fig. 1). This term was also differentiated from the others by being frequently associated with a range of nutritious foods, especially fruit and nuts. Some respondents explicitly noted that snack foods can be either healthy or unhealthy, and some reported that snack foods are typically served in small portion sizes. Convenience was another commonly mentioned attribute.

*Snack food to me means packaged snacks as well, or things like cheese and crackers, popcorn. Snack food can be healthy or unhealthy (F, 25–34, underweight)*

*Snack could be healthy or unhealthy in small proportions and not a major meal (F, 35–44, normal-weight)*

*Something that you have in between meals like a biscuit or apple (F, 45–54, obese)*

*Small food like nuts, dried fruit, muesli bars (F, 45–54, overweight)*

*Bite-sized food, can be healthy or unhealthy (M, 35–44, normal-weight)*

*Convenience food that is quick and easy to eat. It is often unhealthy, but can also be healthy (M, 45–54, normal-weight)*

‘Junk food’ was explicitly noted to be unhealthy by around half (51%) of the sample. Similar to the reaction to discretionary foods, responses were heavily skewed towards health concerns. However, this was apparent across a much larger base of respondents, indicating a much stronger common understanding of the term. There appeared to be a fundamental tension between perceptions of unhealthiness and perceptions of convenience (Fig. 1). Overall, junk food was most clearly distinguished by common references to overall energy content and specific negative nutrients such as fat, sugar, and salt. This type of food was generally seen as being devoid of nutritional value and often associated with fast food restaurants:

*Unhealthy and not good for you. Too many calories (F, 45–54, obese)*

*Food that is convenient, reasonably cheap, and contains large amounts of sodium or sugar (F, 25–34, overweight)*

*Pre-packaged food high in salt and fat (F, 35–44, normal-weight)*

*Food high in fat and/or sugar with little nutritional value (F, 45–54, obese)*

*Highly processed, fat food that taste good (F, 35–44, underweight)*

*Deep fried, with preservatives (M, 35–44, normal-weight)*

*Most fast food outlets (M, 45–54, obese)*

There was occasional acknowledgement that despite all these negative attributes, junk food could be attractive:

*It means food that is not good for you, but sometimes you just need it (M, 25–34, obese)*

*Food that should only be eaten in moderation, but taste good (F, 25–34, overweight)*

*Horrible food that turns your stomach into a garbage dump. And yet, I still eat it! (F, 25–34, obese)*

Party food was the term that produced the broadest range of descriptions, ranging from those referring to the context in which the food was consumed (e.g., special occasions and parties) to those referring to how the foods are physically eaten (e.g., finger food and small portions). This diversity was also reflected in the range of foods provided as examples of party food:

*Food served at a party – fun, easy, playful, and junk (F, 35–44, overweight)*

*Bite size food that guests can pick and choose from, usually a combination of junk food and simple meal type food (M, 35–44, overweight)*

*Food that can be eaten without a knife, fork and plate. It can be picked up in one hand and consumed in small portions (M, 55–64, normal weight)*

*Food that is eaten in celebratory times (F, 45–54, obese)*

*Food that people graze on like chips and lollies (M, 25–34, obese).*

*Food that you’d consume in a social environment that’s small, so you can eat it standing up and moving around (M, 35–44, normal weight).*

*Party food to me means food that is easy to eat, such as finger food, chips, sandwiches, pizza (F, 25–34, underweight)*

As apparent in Fig. 1, the fit with the three dimensions was low overall for the term party foods. This was largely the result of many respondents listing specific types of party foods in response to this food term rather than providing a descriptor of its meaning. In addition, some respondents used the other terms included in the study in their descriptions of this type of food; 33 mentioned junk food and 21 mentioned snack food. This may suggest that this term has a less distinct conceptual profile compared to other terms that are frequently used to describe unhealthy foods.

## Discussion

Previous research in the field of nutrition has highlighted the need to ensure public health messages use language that is of specific relevance to the target audience [8]. The aim of the present study was to gain an appreciation of the colloquial meanings of words used to describe EDNP foods to enhance the effectiveness of healthy eating campaigns. A key finding of the present study was that the term ‘discretionary foods’ had little if any meaning for many respondents. This is of concern given the reliance of the Australian Dietary Guidelines on people being aware of the difference between core and discretionary foods and balancing their diets accordingly [12]. The word ‘discretionary’ was borrowed from the US *MyPyramid* food guide for inclusion in the most recent Australia Dietary Guidelines [12, 16]. Although feedback provided by various stakeholders at the time the new Guidelines were developed included specific recommendations to ensure the general public was informed of the meaning of the term discretionary foods [17], this does not appear to have occurred. The comparable terminology used in the previous version of the Australian Guide to Healthy Eating published in 1998 was ‘extra foods’ [18, 19], which may (or may not) be more meaningful to Australians.

The confusion associated with the term discretionary suggests two possible strategic options: (i) invest in the public education necessary to increase familiarity with the language used in the current Australian Guidelines or (ii) modify future guidelines to use terms that are better understood by the general population. The second option would appear to be the most cost-effective and time-efficient method of achieving congruence between the language used in formal nutrition guidelines and that used among the general population. Specifically, the use of the term junk food in nutrition guidance is likely to represent the surest means of communicating the intended meaning to the Australian public.

A more positive finding was that some respondents referred to healthy snacks, and tended to associate snacking with small portion sizes. While the results indicate that ‘snack food’ currently has both healthy and unhealthy connotations, there may be the potential to co-opt the term to a greater extent over time to make eating between meals synonymous with consuming appropriate amounts of nutritious foods. An example of where this has been done successfully is the Australian Crunch&Sip program that involves primary school children consuming fruit and vegetables during their classes at school to increase their total intake of these foods [20].

Overall, ‘junk food’ was the term that appeared to capture the sentiments most closely aligned with those encompassed by the EDNP category as discussed in the nutrition literature [4]. Respondents associated this term with very unhealthy foods that are high in negative nutrients, without a corresponding evocation of concepts relating to infrequent consumption and small portion sizes that were associated with other terms that could serve to constrain intake. Instead, perceived convenience and ready availability through ubiquitous quick service restaurants appear to at least partially offset concerns about unhealthiness. This suggests that healthy eating campaigns face the challenge of convincing consumers that healthy foods can be quick and easy to prepare, as well as nutritious. The failure of taste to emerge as a meaningful attribute of any of the four terms indicates that efforts to reduce the consumption of unhealthy foods could focus instead on these other aspects relating to nutrition and preparation.

While communications designed to cue people in to the kinds of foods being discussed in healthy eating campaigns may benefit from the term junk food to differentiate a healthy diet from one featuring substantial quantities of unhealthy foods, this is likely to attract the ire of the food industry. Analysts have noted that the food industry increasingly employs similar strategies to the tobacco industry in protecting its interests through corporate social responsibility programs, denial, and litigation [21, 22]. Those implementing social marketing campaigns using this terminology may therefore need to brace themselves from any ensuing pressure from this sector.

The primary limitations of the present study are the use of a web panel, which may not be representative of the broader Australian population, and the short-answer format that prevented the ability to probe for further clarification. However, similar approaches have been used previously effectively to generate findings of relevance to nutrition-related health promotion [23]. Future research could address these methodological limitations in other geographical contexts to assess the extent to which the results pertain to other populations.

## Conclusion

The results of the present study show that different terms for energy dense, nutrient poor (EDNP) foods can have very different meanings for consumers, and these meanings vary little according to demographic characteristics. As such, there is a need to be selective in the use of these terms in public health communications to ensure the intended meaning is being conveyed. The term 'junk food' appears to be the most appropriate term to use in the Australian context to achieve this outcome.

## Abbreviations

BMI: Body mass index
EDNP: Energy dense nutrient poor
F: Female
M: Male
